# Risk Stratification for Atrial Fibrillation and Outcomes in Tachycardia-Bradycardia Syndrome: Ablation vs. Pacing

**DOI:** 10.3389/fcvm.2021.674471

**Published:** 2021-06-08

**Authors:** Rongfeng Zhang, Yue Wang, Minghui Yang, Yiheng Yang, Zhengyan Wang, Xiaomeng Yin, Yingxue Dong, Xiaohong Yu, Xianjie Xiao, Lianjun Gao, Yunlong Xia

**Affiliations:** Department of Cardiology, First Affiliated Hospital of Dalian Medical University, Dalian, China

**Keywords:** atrial fibrillation, tachycardia-bradycardia syndrome, long pauses, catheter ablation, pacing, long outcome

## Abstract

**Background:** Catheter ablation of atrial fibrillation is an alternative treatment for patients with tachycardia-bradycardia syndrome (TBS) to avoid pacemaker implantation. The risk stratification for atrial fibrillation and outcomes between ablation and pacing has not been fully evaluated.

**Methods:** This retrospective study involved 306 TBS patients, including 141 patients who received catheter ablation (Ablation group, age: 62.2 ± 9.0 months, mean longest pauses: 5.2 ± 2.2 s) and 165 patients who received pacemaker implement (Pacing group, age: 62.3 ± 9.1 months, mean longest pauses: 6.0 ± 2.3 s). The primary endpoint was a composite of call cause mortality, cardiovascular-related hospitalization or thrombosis events (stroke, or peripheral thrombosis). The second endpoint was progress of atrial fibrillation and heart failure.

**Results:** After a median follow-up of 75.4 months, the primary endpoint occurred in significantly higher patients in the pacing group than in the ablation group (59.4 vs.15.6%, OR 6.05, 95% CI: 3.73–9.80, *P* < 0.001). None of deaths was occurred in ablation group, and 1 death occurred due to cancer. Cardiovascular-related hospitalization occurred in 50.9% of the pacing group compared with 14.2% in the ablation group (OR: 4.87, 95% CI: 2.99–7.95, *P* < 0.001). More thrombosis events occurred in the pacing group than in the ablation group (12.7 vs. 2.1%, OR 6.06, 95% CI: 1.81–20.35, *P* = 0.004). Significant more patients progressed to persistent atrial fibrillation in pacing group than in ablation group (23.6 vs. 2.1%, *P* < 0.001). The NYHA classification of the pacing group was significantly higher than that of the ablation group (2.11 ± 0.83 vs. 1.50 ± 0.74, *P* < 0.001). The proportion of antiarrhythmic drugs and anticoagulants used in the pacing group was significantly higher than that in the ablation group (41.2 vs. 7.1%, *P* < 0.001; 16.4 vs. 2.1%, *P* = 0.009).

**Conclusion:** Catheter ablation for patients with TBS was associated with a significantly lower rate of a composite end point of cardiovascular related hospitalization and thromboembolic events. Furthermore, catheter ablation reduced the progression of atrial fibrillation and heart failure.

## Introduction

Tachycardia-bradycardia syndrome (TBS) is a common clinical arrhythmia used to describe a special subtype of sick sinus syndrome (SSS), with a long pause (RR intervals > 3 s) on termination of atrial fibrillation (AF) (1, 2). Patients with TBS are at a higher risk of amaurosis, syncope, and even sudden death (3). Guidelines determined that catheter ablation could be used as an alternative treatment for patients with TBS to avoid pacemaker implantation and the evidence recommendation level is IIa (4, 5). However, both treatment options for patients are at potential risk. AF or device related problems may remain even after pacemaker implantation, such as (1) the effect of antiarrhythmic drugs on atrial fibrillation is very limited and the incidences of arrhythmic effects and extracardiac adverse effects are high (6); (2) the incidence of atrial fibrillation-related symptoms, rehospitalization, stroke, progression of atrial fibrillation, and atrial fibrillation-mediated cardiomyopathy persists (7–9); and (3) pacemaker-related complications, such as infections and pacemaker-mediated cardiomyopathy are issues (10).

Previous studies only compared the feasibility and safety between ablation and pacing strategy in TBS patients. Studies showed that >85% of the patients may avoid the pacemaker when ablation for atrial fibrillation was performed. However, the long outcome of ablation for atrial fibrillation superior to pacing is not clear. In our study, we conducted a large-scale retrospective analysis involving 306 patients with TBS with an average follow-up time of 6 years, to evaluate whether catheter ablation improved the long-term outcome of the patients with TBS compared with cardiac pacing.

## Method

### Study Population

This single-center retrospective study was approved by the Ethics Committee of the First Affiliated Hospital of Dalian Medical University, Liaoning Province, China. We retrospectively analyzed 1,371 patients undergoing pacemaker implantation and 795 patients underwent catheter ablation due to atrial fibrillation from 2012 to 2017. A total of 306 patients with TBS were ultimately selected, including 165 patients with pacemaker implantation (Pacing group) and 141 patients with catheter ablation (Ablation group).

**Figure 1:**
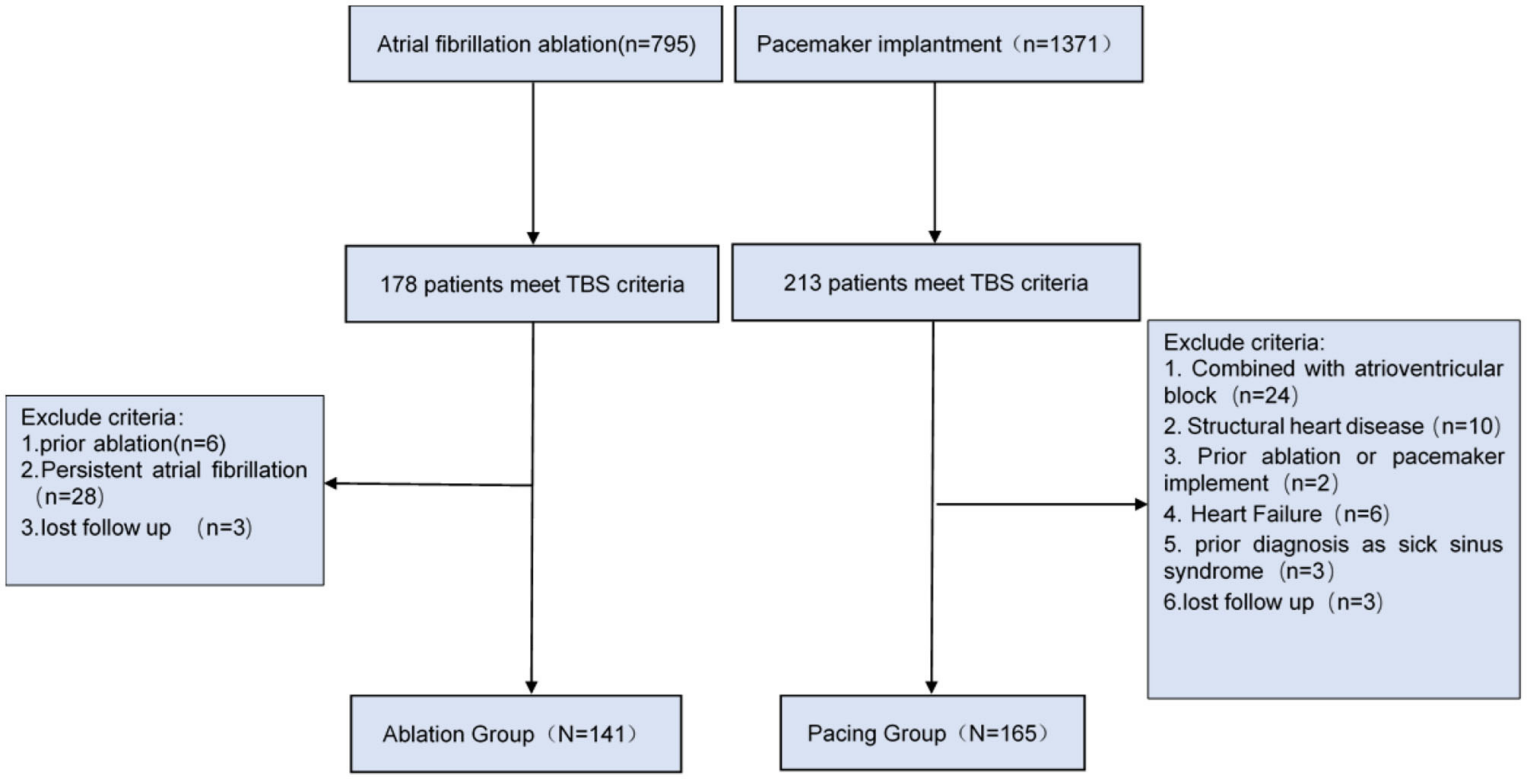
Study population and flow chart.

In this study, TBS diagnosis was in accordance with the diagnostic criteria of BM Kaplan (11), which define TBS as paroxysmal atrial fibrillation, flutter, or tachycardia followed by sinoatrial block or sinus arrest resulting in Stokes-Adams attacks. Patients who had both atrioventricular block/structural heart disease/heart failure, and/or had received radiofrequency ablation or pacemaker implantation in the past were excluded from the study (the screening process is shown in Figure 1).

### Operation Strategy

We provided two treatment options and listed the pros and cons to the patient prior to procedure. And then the patient chose one strategy. In the ablation group, the TBS patients were all diagnosed with paroxysmal atrial fibrillation and received pulmonary vein isolation (PVI) only without addition lesion sets as the ablation strategy. The ablation procedures for this group are as described in a previous study (12). In brief, PV isolation was performed by ablation catheter Navistar Thermocool 3.5-mm D-F curve with Smart Touch technology (Biosense Webster) using contiguous circumferential lesions guided by (lasso™, Biosense and Webster, Inc., CA, USA). RF energy was applied in a power-controlled mode with a power limited of 35 W (30 W at the posterior wall) and a maximal temperature of 45°C. At each point, a radiofrequency current was applied until a voltage of <0.1 mV was achieved, with a maximum of 30 s per point. In the pacing group, the TBS patients all had paroxysmal atrial fibrillation and received DDD or DDDR pacing, and the procedures were as described by Dong et al. (13).

### Study Endpoint

The primary endpoint of this study was a composite endpoint, consisting of all cause mortality cardiovascular rehospitalization and thromboembolic events. Cardiovascular rehospitalization was defined as patients who were re-hospitalized for cardiovascular diseases, including tachycardia, bradycardia, coronary atherosclerotic heart disease (i.e., angina pectoris and/or myocardial infarction), and heart failure. The definition of thrombotic events referred to the occurrence of stroke and/or peripheral thrombotic events (i.e., pulmonary embolism, mesenteric artery embolism, and lower extremity arterial embolism). The definition of the progression of heart failure, we are mainly concerned with NYHA cardiac function grade and left ventricular enlargement or ejection fraction decrease.

### Follow-Up

Patients in the ablation group and the pacing group underwent follow-up for an average of 73.2 ± 17.0 months and 77.6 ± 21.3 months, respectively. The follow-ups were completed by a designated follow-up clinic. Patients in the ablation group had follow-ups in the postoperative months 3, 6, and 12, followed by once every 12 months after the operation via telephone and outpatient visit. Patients in the pacing group were followed up by retrospectively reviewing patient pacemaker programmed records, as well as through telephone and outpatient visits. Twenty-four-hour holter or ECG in the ablation group and pacing group were performed to detect the recurrence of AF at 3, 6, and 12 months visit and annually visit during follow up period. Patient data such as symptoms, recurrence of atrial fibrillation, repeated pacing or catheter ablation, usage of medication, rehospitalization occurrence, and the reasons for cardiovascular rehospitalization or thromboembolic events were collected during follow-ups.

### Statistical Analysis

The continuous variables are presented as mean ± standard deviation and compared using an independent sample *t-*test, and the categorical variables are presented as count and percentage and analyzed using the chi-square test or odds ratio (OR) value. *P* < 0.05 was considered to indicate a significant difference between the groups. The Kaplan–Meier curve was used to compare the incidences of cardiovascular-related rehospitalization, stroke, and/or peripheral thromboembolism, and the log-rank test was used for evaluation. SPSS 23.0 software (IBM Corp., Armonk, NY, USA) was used for statistical analysis in this study.

## Results

### General Characteristics of the Study Subjects

The clinical characteristics of the patients with TBS are shown in Table 1. A total of 306 patients with TBS were selected, including 141 patients in the ablation group, with women accounting for 53.2% and an average age of 62.7 ± 8.8 years, and 165 patients in the pacing group, with women accounting for 52.7% and an average age of 62.4 ± 8.4 years. The longest pauses after termination of atrial fibrillation in the pacing group was slightly longer than that in the ablation group, but this difference was not significant (6.0 ± 2.4 vs. 5.2 ± 2.2 s, *P* = 0.081). The total heart rate per 24 h of the pacing group were lower than that of the ablation group (89,311 ± 19,422 vs. 97,179 ± 16,888, *P* = 0.030), but the average heart rate had no statistically significant difference between the two groups (*P* = 0.283). There was no significant difference in CHA2DS2-VASc score between the two groups (1.65 ± 1.0 vs. 1.75 ± 1.2, *P* = 0.469) (Table 2).

**Table 1:** Characteristics of the study subjects.

	**TBS patients (*n* = 306)**
Female (*n*, %)	162 (52.9%)
Age (mean ± SD, y)	62.6 ± 8.6
Diabetes (*n*, %)	75 (24.5%)
Hypertension (*n*, %)	137 (44.7%)
Coronary heart disease (*n*, %)	61 (19.9%)
Stroke (*n*, %)	5 (1.6%)
AF duration (Mean ± SD, y)	4.6 ± 3.4
Total heart rate (mean ± SD, beats/24 h)	91,486 ± 13,341
Mean heart rate (mean ± SD, beats/min)	64.9 ± 8.4
Longest pause (mean ± SD, s)	5.6 ± 2.3
**Symptom**	
Amaurosis (*n*, %)	111 (36.2%)
Syncope (*n*, %)	97 (31.7%)
LAD (mean ± SD, mm)	38.4 ± 4.18
LVD (mean ± SD, mm)	45.42 ± 4.16
LVEF (mean ± SD, %)	57.7 ± 2.38
CHA_2_DS_2_-VASc score (14)	1.7 ± 1.1
NYHA classification (mean ± SD)	1.4 ± 0.5
Ablation therapy (*n*, %)	141(46%)
Pacing therapy (*n*, %)	165(54%)
**Outcomes**	
Cardiovascular related hospitalization (*n*, %)	104 (34.0%)
Stroke (*n*, %)	18 (5.9%)
Peripheral thrombosis (*n*, %)	6 (2.0%)

**Table 2:** Characteristics of the two groups.

	**Ablation group**	**Pacing group**	***P***
	**(*n* = 141)**	**(*n* = 165)**	
Female (*n*, %)	75 (53.2%)	87 (52.7%)	0.935
Age (mean, y)	62.7 ± 8.8	62.4 ± 8.4	0.790
Diabetes (*n*, %)	30 (21.3%)	45 (27.3%)	0.224
Hypertension (*n*, %)	60 (42.6%)	77 (46.7%)	0.471
Coronary heart disease (*n*, %)	24 (17.0%)	37 (20.6%)	0.238
Stroke (*n*, %)	2 (1.4%)	3 (1.8%)	0.783
Total heart rate (mean ± SD, beats/24 h)	97,179 ± 16,888	89,311 ± 19,422	0.030
Mean heart rate (mean ± SD, beats/min)	65 ± 7	64 ± 8	0.283
AF duration (mean ± SD, y)	4.3 ± 2.96	5.0 ± 3.78	0.065
Longest pause (mean ± SD, s)	5.2 ± 2.2	6.0 ± 2.3	0.081
**Symptom**			
Amaurosis (*n*, %)	47 (33.3%)	64 (38.8%)	0.323
Syncope (*n*, %)	43 (30.5%)	54 (32.7%)	0.676
CHA_2_DS_2_-VASc score (mean ± SD)	1.65 ± 1.0	1.75 ± 1.2	0.469
NYHA classification (mean ± SD)	1.37 ± 0.48	1.45 ± 0.49	0.130
LAD (mean ± SD, mm)	37.96 ± 3.91	38.78 ± 4.37	0.086
LVD (mean ± SD, mm)	45.18 ± 3.86	45.45 ± 4.05	0.553
LVEF (mean ± SD, %)	57.95 ± 2.58	57.52 ± 2.17	0.116

### Comparison of Therapeutic Results Between Ablation Group and Pacing Group

After an average follow-up of 75.5 ± 19.1 months, 116 patients (82.3%) in the ablation group maintained sinus rhythm. In addition, 16 patients (11.4%) in the ablation group had pacemaker implantation due to recurrence of atrial fibrillation with long pauses, and another 6 patients (4.3%) in the ablation group had recurring atrial fibrillation, but no long pauses and without pacemaker implantation. In the pacing group, only 31 patients (18.8%) maintained sinus rhythm, and 8 patients (4.8%) received ablation. Compared with the ablation group, more patients in the pacing group progressed to persistent atrial fibrillation [39 (23.6%) vs. 3 (2.1%), *P* < 0.001], and more patients used antiarrhythmic drugs and anticoagulants [68 (41.2%) vs. 10 (7.1%), *P* < 0.001] and [27 (16.4%) vs. 3 (2.1%), *P* < 0.001]. The New York Heart Association (NYHA) functional classification grade of the pacing group was significantly higher than that of the ablation group (2.11 ± 0.83 vs. 1.50 ± 0.74, *P* < 0.001). According to CHA_2_DS_2_-VASc score, 65 patients in pacing group need long-term anticoagulation therapy, but only 27 people actually insist on oral anticoagulation. 5 patients stopped oral anticoagulants due to bleeding events, 28 patients stopped taking anticoagulants without authorization, and 5 patients took anticoagulants irregularly. Fifty-nine people in ablation group need anticoagulation, but only 3 patients actually insist on oral anticoagulation. Atrial fibrillation was cured in 50 patients, 4 patients personally stopped taking anticoagulants, and 2 patients took anticoagulants irregularly. No significant difference in the incidence of surgery-related complications was found between the ablation and pacing groups (*P* > 0.05; Table 3).

**Table 3:** Comparison of therapeutic results between ablation group and pacing group.

	**Ablation group**	**Pacing group**	***P***
	**(*n* = 141)**	**(*n* = 165)**	
**Symptoms**			
Amaurosis (*n*, %)	9 (6.3%)	0	NS
Syncope (*n*, %)	7 (4.9%)	0	NS
Freedom from AF (*n*, %)	116 (82.3%)	31 (18.8%)	<0.001
AF progression (*n*, %)	3 (2.1%)	39 (23.6%)	<0.001
Heart failure progression (*n*, %)	4 (2.8%)	18 (10.9%)	0.006
NYHA class (mean ± SD)	1.50 ± 0.74	2.11 ± 0.83	<0.001
AADs use (*n*, %)	10 (7.1%)	68 (41.2%)	<0.001
Anticoagulation (*n*, %)	3 (2.1%)	27 (16.4%)	<0.001
Crossover therapy	16	8	0.035
Pacemaker implement (*n*, %)	16 (11.3%)	–	NS
Cather ablation (*n*, %)	–	8 (4.8%)	NS
Operation complications (*n*, %)	2 (1.4%)	4 (2.4%)	0.527

*NS, None Significant*.

### Comparison of Endpoint Between Ablation Group and Pacing Group

**Figure 2:**
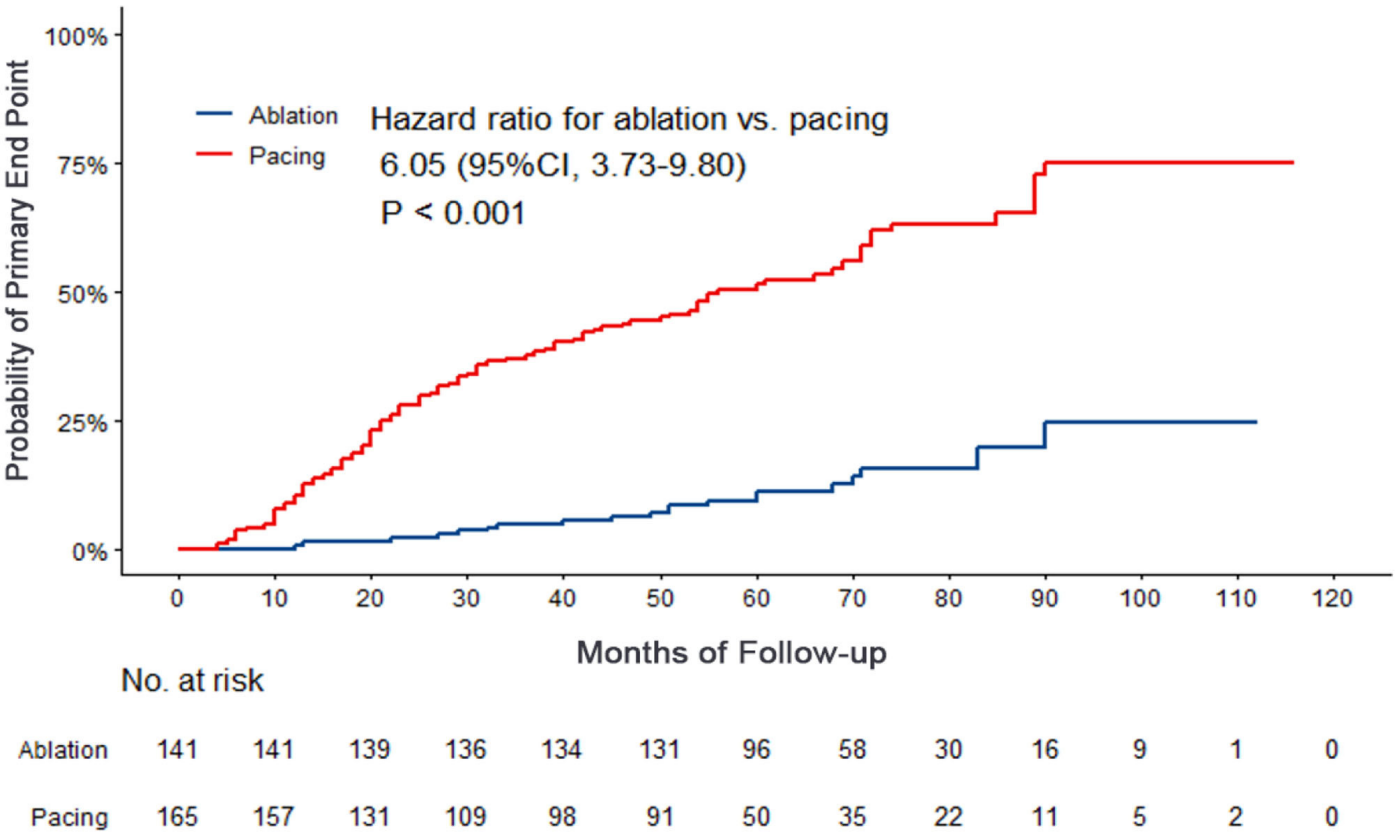
Kaplan–Meier curves comparing probability of the primary end point. Month 0 is the time of the baseline visit. The panel shows the probability of composite end events (cardiovascular related hospitalization or thrombosis events).

**Figure 3:**
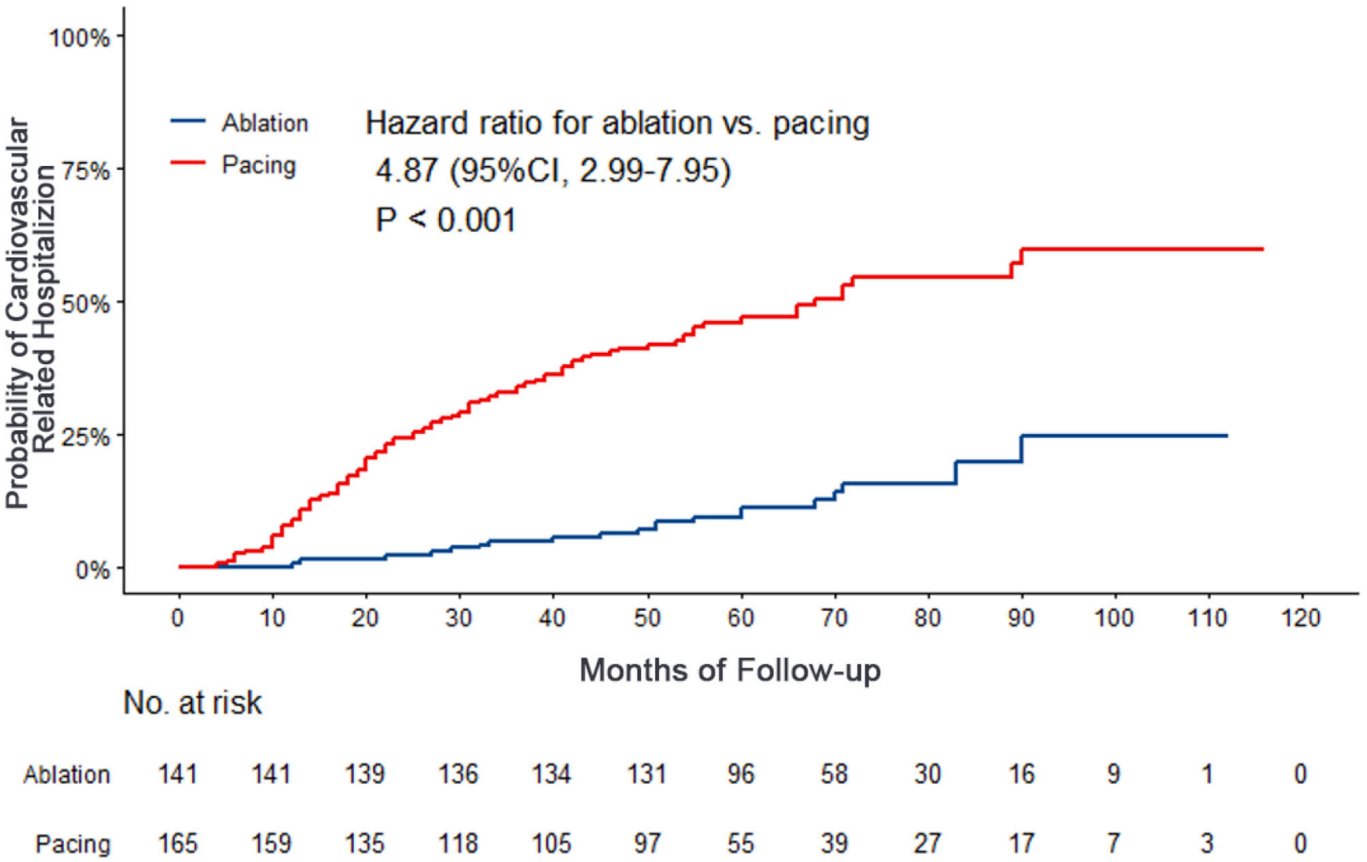
Kaplan–Meier curves comparing probability of cardiovascular related hospitalization. Month 0 is the time of the baseline visit.

**Figure 4:**
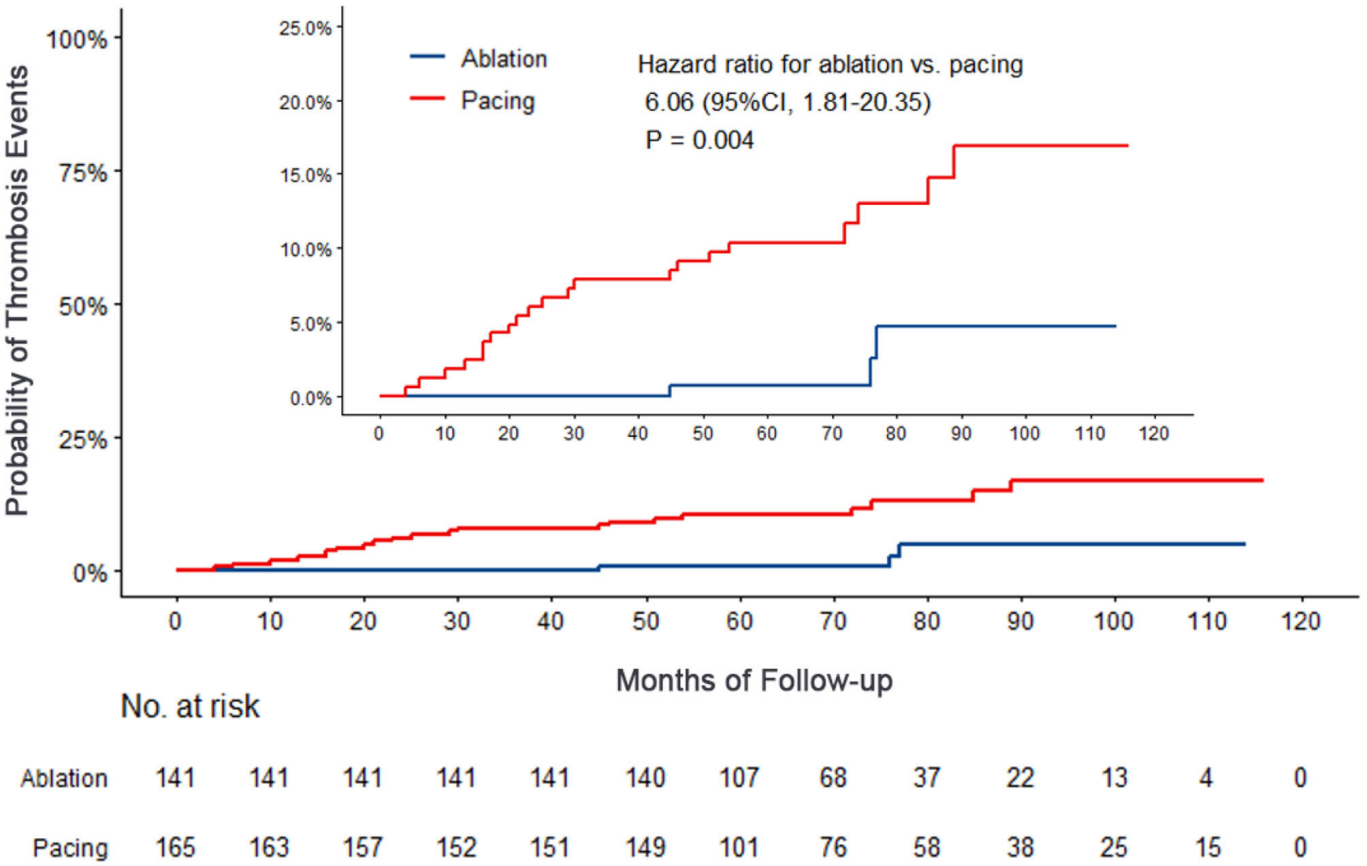
Kaplan–Meier curves comparing probability of thrombosis events. Month 0 is the time of the baseline visit.

Compared with the ablation group, the pacing group had a higher incidence of the primary study endpoint (59.4 vs.14.2%, OR 6.05, 95% CI: 3.73–9.80, *P* < 0.001). The risk of cardiovascular related hospitalization in the pacing group was 4.87-fold that of the ablation group (95% CI: 3.57–11.01, *P* < 0.001). None of deaths was occurred in ablation group, and 1 death occurred due to cancer. A total of 84 cardiovascular-related hospitalization events occurred in the pacing group, primarily due to tachycardia (35.7%), heart failure (27.7%), coronary heart disease (4.2%). There were only 20 cardiovascular-related hospitalization events occurred in the ablation group, which were primarily due to bradycardia (7.8%), tachycardia (2.1%), heart failure (2.1%), and coronary heart disease (2.1%). A total of 9 patients (5.4%) hospitalized due to tachycardia underwent cardioversion therapy. The risk of thromboembolic events in the pacing group was 6.06-fold that of the ablation group (95% CI: 1.81–20.35, *P* < 0.001). A total of 15 strokes and 6 peripheral vascular embolization occurred in the pacing group, while only 3 strokes occurred in the ablation group (Table 4, Figures 2–4). After correcting hypertension, diabetes, stroke history, anticoagulation and CHA2DS2-VASc score by cox regression, there was still significant difference in Primary end point between the two groups (*p* < 0.001).

**Table 4:** Comparison of the primary end point between ablation group and pacing group.

	**Ablation group**	**Pacing group**	**HR (95% CI)**	***P***	***P*-adj**
	***N* (%)**	***N* (%)**			
Primary end point	20 (14.2%)	98 (59.4%)	6.05 (3.73–9.80)	<0.001	<0.001
All-cause mortality	0 (0%)	1 (0.6%)	NS	NS	NS
Cardiovascular related hospitalization	20 (14.2%)	84 (50.9%)	4.87 (2.99–7.95)	<0.001	<0.001
Thrombosis events	3 (2.1%)	21 (12.7%)	6.06 (1.81–20.35)	0.001	0.009
Stroke	3 (2.1%)	15 (9.1%)	4.60 (1.30–16.23)	0.010	–
Peripheral thrombosis	0 (0.0%)	6 (3.6%)	NS	0.022	–

*NS, None Significant*.

## Discussion

### Main Research Findings

This large-scale retrospective study involved 306 patients with TBS, including 141 patients in the ablation group and 165 patients in the pacing group, with an average follow-up of nearly 6 years. The use of ablation for atrial fibrillation in TBS patients was associated with a significantly lower rate of a composite of cardiovascular hospitalization and thrombosis than pacing therapy. Furthermore, catheter ablation reduced the progression of atrial fibrillation and heart failure. To our knowledge, this study was the first to compare the effects of catheter ablation and cardiac pacing on the long-term prognosis in TBS patients, TBS patients may be benefit from ablation therapy vs. pacing therapy.

### Pacing Therapy in TBS

Kaplan and Langendorf were the first to describe TBS in 1973 (11). Patients with TBS often suffer from syncope, syndrome, and even sudden death due to a long pause on termination of atrial fibrillation. Cardiac pacing effectively corrects long pauses after atrial arrhythmia to avoid the occurrence of symptoms, and is recommended by guidelines as the primary treatment plan. However, clinical problems related to atrial fibrillation are still unresolved, and related problems caused by pacing are also occurred. A randomized controlled trial conducted by Lau et al. included 385 patients with paroxysmal atrial fibrillation combined with sinus node dysfunction (SSS), and compared the effects of right atrial appendage pacing and right atrial septum pacing on atrial fibrillation (15). The follow-up of the study lasted 3.1 years, and 25.8% of the patients progressed to persistent atrial fibrillation regardless of the pacing positions and patterns. The Danish Multicenter Randomized trial on single-lead atrial pacing vs. dual-chamber placing in sick sinus syndrome (DANPACE) trial is a randomized controlled trial comparing the effect of single-lead atrial pacemaker (AAIR) pacing or dual chamber pacemaker (DDDR) pacing on long-term prognosis in SSS patients, including 1,348 patients with a follow-up of 5.4 years (16). Of these patients, 25.7% developed atrial fibrillation. In addition, 11.2% of the patients progressed to chronic atrial fibrillation. In our study, 23.6% of the patients progressed to persistent atrial fibrillation, which was consistent with the results of the previous study. In addition, thromboembolic events were a main risk for patients with pacemakers. Brandt et al. (16) conducted a registered trial to evaluate the impact of AAI and DDD pacing modes on rehospitalization or stroke in patients with SSS. In the study the incidence of stroke was nearly 10% regardless of which pacing mode was used. In the present study, the incidence of stroke and peripheral thrombosis was 12.7%. The study conducted by Kristensen et al. (17) showed that atrial fibrillation was an independent risk factor for thrombotic events in patients undergoing pacing (RR: 7.5, 95% CI: 1.6–36.2, *P* = 0.01). Oral anticoagulants are an effective strategy to prevent thromboembolism. However, in this study, only 16.4% of our patients received anticoagulation therapy, but these patients had a relatively high CHA_2_DS_2_-VASC score. Insufficient anticoagulation may be one of the reasons for the high risk of thrombotic events. In fact, there are still challenges in requiring strict anticoagulation therapy for these patients. For example, bleeding, pacemaker pocket infections, and patient compliance may be the primary concerns. For patients with TBS undergoing pacemaker implantation, it is inevitable that anticoagulation, rhythm control, and ventricular rate control of atrial fibrillation will need to be addressed again.

### Catheter Ablation for Atrial Fibrillation in TBS Patients

Numerous studies have confirmed that catheter ablation is an effective and safe method for treating paroxysmal atrial fibrillation, with a success rate of >82% in >5 years (18). A retrospective study conducted by Hada et al. included 65 patients with SSS and atrial fibrillation (SSS-AF). After an average of 1.4 ablations, the patients underwent a 3-year follow-up, showing a success rate of 80.6% (19). Osaka et al. also conducted a study of catheter ablation in patients with SSS-AF and pacemaker implantation (*n* = 51, followed up for 5 years) (20). The success rate of catheter ablation in the study was 86.3%. Inada et al. (21) performed a study was to define the potential role of successful ablation in patients with TBS. During the 5.8 years (range: 5–8.7 years) follow up, 86% patients remained free from AF after the last procedure. Only 8% patients required pacemaker implantation of the study by Chen et al. (22) evaluated the effectiveness and safety of catheter ablation in patients with TBS. Although only 43 patients with TBS were included in the study, 41 (95.3%) patients, who underwent follow-up for 20 months, did not require pacemaker implantation. Kim et al. (23) conducted a larger sample retrospective study involving 121 patients with TBS and followed up for 20 months. They found that 90.9% of the patients with TBS did not need pacemaker implantation. In this study, 88.6% of the patients with TBS undergoing catheter ablation avoided pacemaker implantation, suggesting that catheter ablation can prevent nearly 90% of the patients with TBS from requiring an implanted pacemaker.

### Should Catheter Ablation Be the First Line Therapy for TBS Patients?

Cardiovascular related hospitalization, thromboembolic events, and heart failure progression are essential endpoints for evaluating the prognosis of TBS patients. However, few studies have evaluated the hard endpoints of catheter ablation vs. pacing in patients with TBS. Chen et al. (22) showed that catheter ablation significantly reduced the rate of rehospitalization related to atrial fibrillation. However, no significant difference in cardiovascular rehospitalization rate was found, which may be due to the small sample size of that study. One of our previous studies (24) showed that catheter ablation significantly reduced the risk of new strokes in patients with TBS compared with the pacing group (15.1 vs. 5.4%, *P* < 0.05). In this study, after 5.9 years of follow-up, the risk of cardiovascular-related hospitalization and thrombosis in the TBS patients undergoing cardiac pacing was 6.05-fold higher than that of the patients undergoing catheter ablation (95% CI: 3.73–9.80, *P* < 0.001). The risk of cardiovascular related hospitalization of the pacing group was 4.87-fold than that of the ablation group (95% CI: 2.99–7.95, *P* < 0.001), and the thromboembolic event risk of the pacing group was 6.06-fold than that of the ablation group (95% CI: 1.81–20.35, *P* = 0.001). The long-term outcome data suggested that catheter ablation significantly reduced cardiovascular-related hospitalizations, strokes, and peripheral thromboembolic events, and also effectively reduced atrial fibrillation burden and heart failure progression. Our findings supported that pacing may be a risk factor for worse prognosis in TBS patients. In this cohort, patients received the RV pacing. Considering the impact of right ventricular pacing on heart function, the results may be bias. Recent years, His bundle pacing (HBP) or left bundle branch pacing can achieve the physiological pacing via directly stimulating the His-Purkinje conduction bundle, which can significantly reduce pacing induced cardiomyopathy (25). Future evidence was needed to verify the results in the TBS patients.

### Limitations

This was a retrospective study, so the clinical evidence level is low, and a prospective randomized controlled study is needed to verify our findings. TBS patients in the pacing group had a higher proportion of anticoagulation but inadequate, suggesting that an increase in anticoagulation rate may effectively reduce the incidence of thromboembolic events in the pacing group. Lower use of anticoagulation in the entire group may limit the applicability of the data. Adequate use of anticoagulation in TBS patients may reduce the difference of the prognosis between the two therapy strategies. In our series these patients had relatively normal left atrial size. This data may not be applicable to patients with moderate or severely dilated left atria. Additionally, there may have been a selection bias during the selection of treatment strategies in this retrospective study.

## Data Availability Statement

The original contributions presented in the study are included in the article/supplementary material, further inquiries can be directed to the corresponding author/s.

## Ethics Statement

The studies involving human participants were reviewed and approved by the ethics committee of First Affiliated Hospital of Dalian Medical University. Written informed consent for participation was not required for this study in accordance with the national legislation and the institutional requirements.

## Author Contributions

YX, LG, and XYi contributed to conception and design of the study. YW, ZW, and MY organized the database. XYu and XX performed the statistical analysis. YW wrote the first draft of the manuscript. RZ wrote sections of the manuscript. All authors contributed to manuscript revision, read, and approved the submitted version.

## Conflict of Interest

The authors declare that the research was conducted in the absence of any commercial or financial relationships that could be construed as a potential conflict of interest. The handling editor declared a past co-authorship with one of the authors YX.

## Abbreviations

AAD: Antiarrhythmic drug
AVN: Atrioventricular node
AF: Atrial fibrillation
AUC: Area-under-the-curve
CI: Confidential interval
HF: Heart failure
LAD: Left atrial diameter
LVD: Left ventricular diameter
LVEF: Left ventricular ejection fraction
NOAC: New oral anticoagulants
NYHA: New York Heart Association
TBS: Tachycardia-bradycardia syndrome
Vit K: vitamin K.
